# Establishing extracorporeal membrane oxygenation team increased number of patients and improved data recording

**DOI:** 10.1186/s40560-019-0366-4

**Published:** 2019-02-07

**Authors:** Atthasit Komindr, Ryuzo Abe, Yoshihisa Tateishi, Yuka Takahashi, Jun Goto, Keita Wada, Yutaka Furukawa, Atsushi Sugiura, Taro Imaeda, Natsumi Suga, Noriyuki Hattori, Shigeto Oda

**Affiliations:** 1 Department of Emergency and Critical Care Medicine, Chiba University Graduate School of Medicine, Chiba, Japan; 2 Department of Cardiology, Chiba University Graduate School of Medicine, Chiba, Japan; 3 Emergency Department, King Chulalongkorn Memorial Hospital, Thai Red Cross Society, 35/188 Phayathai, Rachatevee, Bangkok, 10400 Thailand

**Keywords:** Extracorporeal membrane oxygenation, Extracorporeal cardiopulmonary resuscitation, Multidisciplinary team, ECMO team, Data recording

## Abstract

**Background:**

For patients treated with extracorporeal membrane oxygenation (ECMO), employing a well-coordinated, multidisciplinary team specializing in ECMO has reportedly been effective in delivering better clinical outcomes. This study aims to assess the impact of establishing such a specialized team for patients treated with ECMO.

**Method:**

This retrospective cohort study was performed at a tertiary-care hospital in Japan. We reviewed medical records of all consecutive patients treated with ECMO during October 2010–September 2016. The results obtained in pre-ECMO team cases (PRE group; October 2011–September 2012) and post-ECMO team cases (POST group; October 2014–September 2015) were compared.

**Results:**

The results obtained in pre-ECMO team cases (PRE group; October 2011–September 2012) and post-ECMO team cases (POST group; October 2014–September 2015) were compared. During the study period, 177 patients were treated with ECMO. Before the introduction of ECMO team, an average of 22.7 patients underwent ECMO treatment per year; after establishing ECMO team, this number increased to 36.3 patients per year. ECMO was applied mainly to cardiac arrest patients 52/69 (75%). The PRE (*n* = 27) and POST (*n* = 42) groups did not differ with regard to the survival rate to hospital discharge, ECMO duration, ventilator days, and length of hospital stay. However, PaO_2_ and positive end-expiratory pressure were significantly higher in the POST group at 6 h after ECMO initiation than those in the PRE group [367 (186–490) vs. 239 (113–430) mmHg, *p* = 0.047 and 8 (5–10) vs. 7 (5–8) cmH_2_O, *p* = 0.01, respectively]. In addition, data recording the detailed time points of ECMO initiation was conducted in significantly more cases in the POST group (28/126 (22%) than in the PRE group (6/81 (7%); *p* = 0.01).

**Conclusions:**

Following the establishment of an ECMO team, the survival rate of patients treated with ECMO, ECMO duration, and length of hospital stay were not improved. However, the number of ECMO cases increased and the recording of clinical data was improved.

## Background

Extracorporeal membrane oxygenation (ECMO) is a rescue procedure involving the use of a centrifugal pump and an artificial lung applied to critically ill patients. Recent evidences show that ECMO’s indication has been extended to patients with a variety of conditions [1–3], including not only cardiogenic shock and acute respiratory distress syndrome (ARDS), but also cardiac arrest and septic shock [2, 3]. As a result, an increasing number of patients have been treated with ECMO [4].

The clinical outcomes of patients treated with ECMO widely varied among the hospitals, depending on the infrastructure and institutional experience in treating with ECMO [5], even though the ideal institutional requirements for an effective use of ECMO were clearly specified in the guidelines issued by Extracorporeal Life Support Organization and American Thoracic Society [6, 7]. Reportedly, better clinical outcomes can be achieved in patients treated with ECMO with a well-coordinated, multidisciplinary team specializing in ECMO [8]. Owing to a broad range of patient diagnoses, a multidisciplinary team consisting of many specialties of physicians, nurses, clinical engineers, perfusionists, and respiratory therapists has to smoothly cooperate and elucidate the underlying pathophysiology and mechanisms of ECMO. In a practical aspect, clinical settings with such a team approach not only prevents technical errors and develops better treatment strategies but also helps conduct educational and clinical research programs [9]. Successful outcomes can be achieved with well-coordinated teamwork, even with time constraints in stressful situations of ECMO initiation and management.

Nonetheless, the effectiveness of ECMO team intervention on clinical outcomes has not yet been clarified in the literature. Therefore, we hypothesized that the establishment of an ECMO team would improve the clinical outcomes of patients treated with ECMO. Thus, this study aimed to identify the clinical consequences of establishing and implementing an ECMO team.

## Methods

This is a retrospective cohort study, conducted at Chiba University Hospital (an 850-bed, tertiary-care, academic medical center), Chiba prefecture, Japan. The project received ethical approval from the Chiba University Ethics Review Board.

### Study population and data collection

We reviewed medical records of all consecutive patients admitted to the ICU October 2010–September 2016, and those who underwent ECMO were enrolled in the study.

We collected patient demographics data, indications for ECMO, time of ECMO initiation, and outcomes at ECMO removal, in ICU, and at hospital discharge. Additionally, we recorded ventilator settings, results of blood gas analyses, and blood lactate levels before and after ECMO treatments. ECMO use was recorded after one of three indications: cardiac, respiratory, or cardiopulmonary resuscitation (CPR).

Data completion rate was then evaluated using four categories: time to start and stop ECMO, defined as time starting or stopping of the centrifugal pump to create blood circulation into the ECMO circuit; detailed records of time phases involved with ECMO initiation; arterial blood gas (ABG) analysis along with lactate blood levels; and activated clotting time (ACT) at 0, 6, and 24 h after ECMO initiation. ACT was measured using Hemochron Response (Accriva Diagnostics, MA) and a test tube (HRFTCA510), according to the manufacturer’s instruction. Detailed time phases involved with ECMO initiation were expressed as percentages of the following recorded time points: time of deciding ECMO initiation, insertion of a sheath introducer, cannulation initiation, cannulation completion, circuit priming initiation, and circuit priming completion. The data sheets to record these time processes had been already utilized before study period. In addition, ventilator, ICU, and hospital days were also recorded. Lactate clearance was calculated by the equation: [(lactate_initial_ − lactate_delayed_)/lactate_initial_] × 100 (%), for which lactate_initial_ was measured at the start of ECMO and lactate_delayed_ was measured after 6 and 24 h after initiating ECMO treatment.

### ECMO team

Before establishing ECMO team, we had already performed ECMO on > 200 patients with cardiac and/or respiratory failure since 1993. Therefore, all attending doctors in the department of emergency and critical care medicine of our hospital had experience in deciding indication for ECMO, cannulating, initiating, and managing ECMO treatment. In addition, at least one ECMO team member is always on shift of the emergency department and of the intensive care unit. When more than one patient needs to start ECMO simultaneously, other ECMO team members are called. Though not only ECMO team members, but also other doctors and nurses are involved in ECMO treatment, ECMO team members always conduct management of ECMO, including following daily activities: (1) twice daily clinical conference discussing the therapeutic strategy for the patients, (2) morning and evening rounds of all ECMO patients to check mechanical complications, (3) deciding indication for ECMO, cannulating and initiating in all ECMO cases, and (4) weekly conference to review all ECMO cases. Since many ER and ICU nurses and clinical engineers had also been trained to prime ECMO circuit, at least one of them was always on shift during the study period. The ratio of patient to nurse in the ICU was kept 1:1 before and after ECMO team establishment. After launch of ECMO team, all nurses who were in charge of ECMO patient had been trained regularly by an educational program, which included didactic session, water drill, scenario-based simulation, and mortality and morbidity conference at least every 6 months, though in the period before ECMO team they were not trained in a fixed program. The ECMO team was formally established in October 2013 and comprised of emergency and critical care physicians, cardiologist, ER and ICU nurses, and clinical engineers. All the members had experienced numerous ECMO cases and were trained in ECMO treatments. The ECMO team played a significant role in conducting procedures and management of ECMO, as well as in staff training and data collection.

### ECMO treatments

In our institution, ECMO is initiated in accordance with specific criteria, which include cardiac, respiratory, and CPR indications, as follows:

1. Mean arterial blood pressure (MAP) < 60 mmHg due to low cardiac output, sustained after appropriate fluid, inotropes, and vasopressors administration.
2. P/F ratio < 80 due to respiratory dysfunction, under mechanical ventilatory conditions in airway pressure release ventilation mode and nitric oxide inhalation.
3. In-hospital or out-of-hospital cardiac arrest, which was witnessed and whose electrocardiogram result was other than asystole.

However, patients who fulfilled the following exclusion criteria were excluded from an ECMO indication: ≥ 80 years old, with malignancy, or with evidence of severe brain damage. Regardless of whether a veno-arterial (V-A) or veno-venous (V-V) approach was employed, ECMO was performed using a centrifugal pump (CAPIOX® emergency bypass system, Terumo, Tokyo, Japan or Rotaflow® system, Maquet, Rastatt, Germany) and a membrane oxygenator (CAPIOX (LX)®, Terumo or BIOCUBE® 6000, Nipro, Osaka, Japan). The size and type of cannulae were selected at the physician’s discretion (16 to 25 Fr., TOYOBO; Flexmate®, TOYOBO, Osaka, Japan; Bio-Medicus®, Medtronic, Minneapolis, MN, USA; or CAPIOX (X)®, Terumo). During the entire study period, the ECMO pump, oxygenator, vascular catheter, and cannulation technique were kept constant. Initiation of ECMO treatment and catheter cannulation was decided by physicians in the Department of Emergency and Critical Care Medicine. Percutaneous peripheral cannulation was majorly employed among all the vascular access techniques used in the study population. Cannulation was performed by physicians of the department of emergency and critical care medicine, using ultrasound-guided puncture, dilation through guidewire and placement of cannula. Positions of guidewire and cannula were confirmed by mobile X-ray imaging. ECMO circuit was set up by a physician, nurse, or clinical engineer, those who had been already trained in the educational program. In cases of cardiac arrest, body temperature after return of spontaneous circulation (ROSC) was kept below 35.0 °C at least 24 h and rewarmed at the rate of 1 °C/24 h. According to results of electrocardiogram and ultrasonic cardiogram, majority of patients after ROSC underwent coronary angiography and intervention. During ECMO treatment, ECMO blood flow was controlled based on the patient’s circulatory and respiratory conditions, aiming a MAP target of > 60 mmHg, O_2_ saturation of > 90%, and PaCO_2_ of < 50 mmHg. Unfractionated heparin as an anticoagulant was continuously administered and manipulated to achieve the targeted ACT of 180 s and activated partial thromboplastin time (aPTT) between 50 and 75 s. ACT was measured every 2 h and aPTT was measured every 6 h. Platelet count was also measured every 6 h, and platelet concentrate was transfused when it became below 50,000 cm^3^.

### Outcomes between the groups

We first examined annual changes in the number of ECMO cases and their survival rates, followed by a comparison of outcomes before and after ECMO team establishment. We then extracted data on the two groups of patients, before [pre-ECMO team cases (PRE group)] and after [post-ECMO team cases (POST group)] ECMO team establishment. The cases in the PRE group were drawn from October 2011 to September 2012, and those from the POST group from October 2014 to September 2015. The ECMO team officially launched in October 2013; however, even before the establishment, some members already had executed the same roles in ECMO management and staff education. With an increasing number of ECMO patients, the members’ role had been gradually expanded, especially since 2010. Even before officially establishing the ECMO team, the activities of its members in 2013 were almost similar to those in 2014, after the ECMO team formation. Therefore, we included patients from 1 year before and after the formal launch of the ECMO team, as the comparison between patients in 2013 and 2014 was not appropriate to evaluate the effects of the ECMO team.

The primary outcome was the survival rate, and the secondary outcomes were the number of ECMO cases and the completion rates of data recording, which was defined as the percentage of data recorded, as described above.

### Statistical analysis

Clinical data are presented as medians with interquartile ranges (IQR) for continuous data, and as frequencies and percentages for categorical data. Univariate analysis was performed using a standard statistical package (SPSS Statistics for Windows, Version 22.0; IBM Corp, Armonk, New York). Comparison of univariate continuous data was performed using Mann–Whitney *U* tests, and categorical data were compared using Fisher’s extract test. Statistical significance was set for comparisons with *p* < 0.05.

## Results

### Study population

**Fig. 1:**
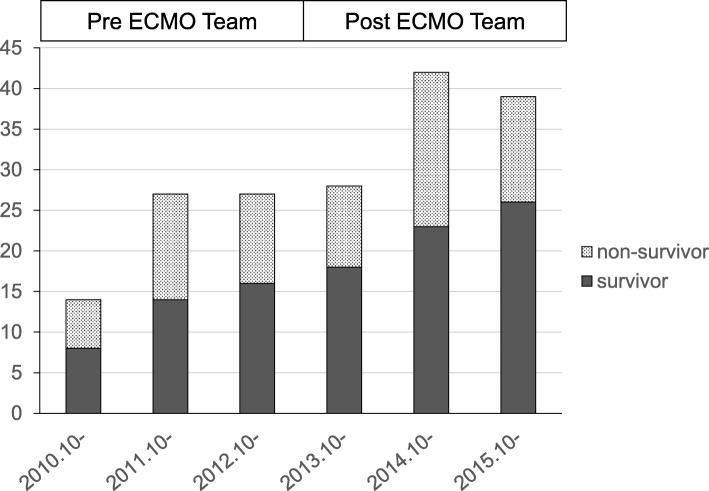
Annual changes in number of patients treated with ECMO, before and after ECMO team establishment. Pre-ECMO team period, from October 2010 to September 2013; post-ECMO team period, from October 2013 to September 2016. Survivor, patients who were discharged alive from the hospital. Non-survivor, patients who died in the hospital; ECMO, extracorporeal membrane oxygenation

During the study period, of the 10,875 patients admitted to the ICU, 177 were treated with ECMO, and their survival rate to hospital discharge was 59.3% (105/177) (Fig. 1). Of these, 68 underwent ECMO treatment within 3 years (22.7/year) before the establishment of the ECMO team in October 2013, and 109 were treated over the 3 years (36.3/year) after the ECMO team establishment. Interestingly, the survival rates between these two groups were not significantly different (38/68 or 55.9% and 67/109 or 61.5%, respectively).

Subsequently, data extraction and comparison of patients before and after the ECMO team establishment was performed, with a total of 69 (27 in PRE and 42 in POST group) patients included in this comparison study cohort.

### Comparison between PRE and POST groups

Patients’ baseline demographic information is shown in Table 1. The median age was 61 years, and 46 (67%) were male. ECMO treatment was initiated via a veno-arterial approach in 65 (94%) patients; for 52 (75%) patients with cardiac arrest, the treatment was performed as extracorporeal cardiopulmonary resuscitation (ECPR). The APACHE II score before ECMO treatment, ECMO type, ECMO indication, site of ECMO initiation, and interventions during ECMO were not different between the POST and PRE groups.

**Table 1:** Patient demographics and baseline characteristics

	All patients (*n* = 69)	Pre-ECMO team (*n* = 27)	Post-ECMO team (*n* = 42)	*p* value
Age in years, median (IQR)	61 (42–74)	61 (42–77)	62 (35–73)	0.60
Male, *n* (%)	46 (67%)	19 (70%)	27 (64%)	0.60
APACHE II score, median (IQR)	36 (29–40)	38 (32–41)	35 (27–39)	0.14
ECMO type, *n* (%)				
ECMO type, *n* (%)				
V-A	65 (94%)	25 (93%)	40 (95%)	0.64
V-V	4 (6%)	2 (7%)	2 (5%)	
Cause of ECMO, *n* (%)				
Cardiac arrest	52 (75%)	20 (74%)	32 (76%)	0.90
Cardiac (excluding cardiac arrest)	13 (19%)	5 (19%)	8 (19%)	
Respiratory	4 (6%)	2 (7%)	2 (5%)	
Place to start ECMO, *n* (%)				
ED	21 (31%)	8 (30%)	13 (31%)	0.39
ICU	22 (32%)	7 (26%)	15 (36%)	
OR	5 (7%)	4 (15%)	1 (2%)	
Other*	14 (20%)	5 (18%)	9 (21%)	
Referral hospital	7 (10%)	3 (11%)	4 (10%)	
Interventions during ECMO, *n* (%)				
CAG	30 (44%)	12 (44%)	18 (43%)	0.90
IABP	30 (44%)	15 (56%)	15 (36%)	0.11
CRRT	31 (45%)	13 (48%)	18 (43%)	0.67

*IQR* interquartile range, *APACHE* acute physiology and chronic health evaluation, *ECMO* extracorporeal membrane oxygenation, *V-A* veno-arterial, *V-V* veno-venous, *ED* emergency department, *ICU* intensive care unit, *CAG* coronary angiogram, *IABP* intra-aortic balloon pump, *CRRT* continuous renal replacement therapy

*Operation room, catheterization laboratory, computed tomography room, general ward, and referral hospital

Regarding the primary outcome of survival to hospital discharge, the two groups showed no significant difference (PRE group, 16/27 (59%) vs. POST group 30/42 (71%), *p* = 0.30, OR 1.72; 95% CI, 0.62–4.76), as detailed in Table 2. In addition, survival to wean off ECMO, survival to ICU discharge, and survival at day 28 regardless of discharge were also not significantly different between the two groups.

**Table 2:** Comparison of survival outcome between pre-ECMO team and post-ECMO team established

	All patients (*n* = 69)	Pre-ECMO team (*n* = 27)	Post-ECMO team (*n* = 42)	OR	95% CI for OR		*p* value
					Lower	Upper	
Survival to hospital discharge	46 (67%)	16 (59%)	30 (71%)	1.72	0.62	4.76	0.30
Survival to ICU discharge	43 (62%)	16 (59%)	27 (64%)	1.24	0.46	3.34	0.67
28-day survival	36 (52%)	14 (52%)	22 (52%)	1.02	0.39	2.69	0.97
Survival to wean off ECMO	32 (46%)	13 (48%)	19 (45%)	0.89	0.34	2.35	0.81

In addition, ECMO duration, ventilator days, and length of hospital stay among the patients who survived did not differ (Table 3). However, at 6 h after ECMO initiation, PaO_2_ and positive end-expiratory pressure (PEEP) were significantly higher in the POST group than those in the PRE group [367 (186–490) vs. 239 (113–430) mmHg, *p* = 0.047 and 8 (5–10) vs. 7 (5–8) cmH_2_O, *p* = 0.01, respectively]. Other ABG values, ACT, lactate level, and lactate clearance rate as well as ventilator settings and ECMO flow at 6 and 24 h after ECMO initiation showed no difference between the PRE and POST groups.

**Table 3:** Comparison of clinical outcome and laboratory data between the pre-ECMO team and post-ECMO team established

	All patients (*n* = 69)	Pre-ECMO team (*n* = 27)	Post-ECMO team (*n* = 42)	*p* value
Time from cannulation to ECMO started (min), median (IQR)	15 (6.5–32.5)	16 (15–52)	15 (6–32)	0.37
Duration of ECMO (days)	3 (1–4)	3 (1–4)	3 (2–5)	0.65
Length of mechanical ventilation (days)	10 (6–16)	11 (7–23)	8 (5–14)	0.21
Length of ICU stay (days)	16 (9–23)	18 (9–30)	16 (8–20)	0.17
Length of hospital stay (days)	51 (25–76)	55 (27–125)	51 (22–70)	0.30
Before ECMO, median (IQR)				
PH	7.22 (7.05–7.38)	7.19 (7.03–7.44)	7.23 (7.07–7.36)	0.86
PaCO_2_ (mmHg)	38 (48–68)	47 (40–87)	49 (37–66)	0.39
PaO_2_ (mmHg)	103 (55–169)	85 (41–121)	122 (75–246)	0.13
HCO_3_ (cmH_2_O)	21 (15–26)	22 (15–28)	20 (15–25)	0.28
Lactate (mmol/L)	9.9 (4.1–14.15)	7.7 (3.4–15.5)	10.5 (5.3–13.8)	0.88
F_i_O_2_	0.6 (0.5–1.0)	0.8 (0.6–1.0)	0.6 (0.50–1.00)	0.57
PEEP (cmH_2_O)	8 (5–10)	9 (6–10)	8 (4–10)	0.74
On-ECMO 6 h, median (IQR)				
PH	7.42 (7.34–7.50)	7.44 (7.35–7.51)	7.41 (7.33–7.49)	0.47
PaCO_2_ (mmHg)	32 (27–39)	31 (25–36)	33 (27–40)	0.36
PaO_2_ (mmHg)	322 (148–470)	239 (113–430)	367 (186–490)	0.047
HCO_3_ (cmH_2_O)	21 (18–24)	20 (16–26)	22 (18–24)	0.71
Lactate (mmol/L)	5.8 (4.4–9.1)	5.9 (4.7–8.7)	5.8 (3.4–9.8)	0.74
F_i_O_2_	0.5 (0.4–0.6)	0.6 (0.5–0.7)	0.5 (0.4–0.6)	0.38
PEEP (cmH_2_O)	8 (5–10)	7 (5–8)	8 (5–10)	0.01
ACT (seconds)	187 (165–207)	201 (168–214)	185 (160–202)	0.23
ECMO blood flow (L/min)	2.2 (1.9–2.8)	2.2 (2.0–3.0)	2.2 (1.8–2.6)	0.36
On-ECMO 24 h, median IQR				
PaO_2_/F_i_O_2_	462 (264–672)	438 (208–598)	469 (299–740)	0.18
PH	7.43 (7.38–7.52)	7.44 (7.38–7.55)	7.42 (7.37–7.51)	0.26
PaCO_2_ (mmHg)	36 (29–40)	33 (27–39)	36 (30–42)	0.30
PaO_2_ (mmHg)	221 (133–322)	210 (125–274)	246 (136–350)	0.28
HCO_3_ (cmH_2_O)	24 (22–27)	25 (19–29)	24 (22–27)	0.62
Lactate (mmol/L)	2.8 (1.9–4.4)	3.2 (2.8–4.9)	2.6 (1.3–4.4)	0.19
F_i_O_2_	0.4 (0.4–0.6)	0.5 (0.4–0.6)	0.4 (0.4–0.5)	0.08
PEEP (cmH_2_O)	8 (6–10)	8 (5–10)	8 (7–10)	0.57
ACT (seconds)	182 (161–196)	182 (160–196)	186 (163–197)	0.54
ECMO blood flow (L/min)	2.2 (1.7–3.0)	2.3 (1.9–3.0)	2.2 (1.7–3.1)	0.63
Lactate clearance, median (IQR)				
At 6 h (%)	15 (−27 to 54)	4 (−51 to 57)	18 (−17 to 54)	0.59
At 24 h (%)	61 (18 to 80)	51 (−20 to 83)	61 (18 to 80)	0.71

*ECMO* extracorporeal membrane oxygenation, *IQR* interquartile range, *PEEP* positive end-expiratory pressure, *ACT* activated clotting time

While comparing the data recorded, detailed time process of ECMO initiation was completely recorded in significantly more number of cases in the POST group (28/126, 22%) than in the PRE group (6/81, 7%), *p* = 0.01. However, the completion rate of recording ECMO start and stop times was not different (PRE; 50/54, 93% and POST; 81/84, 96%), *p* = 0.20 as shown in Table 4. All values for ABG, lactate, and ACT at 0, 6, and 24 h were completely recorded in a similar proportion of cases in the two groups.

**Table 4:** Comparison of completion rate of data recording between the pre- and post-ECMO teams

	All patients (*n* = 69)	Pre-ECMO team (*n* = 27)	Post-ECMO team (*n* = 42)	*p* value
Detailed time process of ECMO initiation, *n* (%)	34/207 (16%)	6/81 (7%)	28/126 (22%)	0.01
Time to start and stop ECMO, *n* (%)	134/138 (97%)	50/54 (93%)	81/84 (96%)	0.20
ABG with lactate at 0, 6, and 24 h, *n* (%)*	154/192 (80%)	57/75 (76%)	97/117 (83%)	0.31
ACT at 6 and 24 h, *n* (%)*	103/114 (90%)	35/42 (83%)	68/72 (94%)	0.05

*ECMO* extracorporeal membrane oxygenation, *ABG* arterial blood gas, *ACT* activated clotting time

*Total number of data was missing due to weaned-off ECMO or died before 6 or 24 h. Detailed time process of ECMO initiation was expressed as percentage of following time points recorded; time of decision to start ECMO, insertion of sheath introducer, cannulation started, cannulation finished, circuit priming started, and circuit priming finished. Data sheet to input these pieces of information was made before the study period

## Discussion

In the present study survival rates to hospital discharge did not differ between patients treated with ECMO before and after the establishment of the ECMO team. However, the number of ECMO cases increased, and data recording during ECMO treatment improved. Most previous studies regarding ECMO teams have reported the experiences involving ECMO team inauguration and have examined the optimal structure required for team specialized for performing ECPR or transporting ECMO patients [10, 11]. However, the clinical impact of the team approach had not been reported, except for a study by Su et al., which noted a reduced time to start ECPR after relevant training via a simulation program [12].

Recently, Na et al. reported clinical outcomes in patients with severe acute respiratory failure, who were treated with ECMO before and after ECMO team implementation [13]. They reported that mortality was lower and successfully weaned-off ECMO rate was higher in patients treated in the post-ECMO team period than those treated in the pre-ECMO team period. They also reported that the incidence of cannula problems was reduced after ECMO team implementation. However, in the present study, the survival rate, as the primary outcome, did not improve after ECMO team establishment. This could have been caused by several factors, listed below: (1) Background of study population: Most patients in our investigation had experienced cardiac arrest and were resuscitated using ECPR, whereas all patients enrolled in a retrospective study of ARDS patients were treated with V-V ECMO [13]. Even if the quality of ECMO management was improved by the specialized team-based approach, the mortality of patients with cardiac arrest could not be improved. Additionally, there is a possibility that an ECMO team is specifically effective for patients with ARDS treated with V-V ECMO because such patients tend to need longer ECMO support than those with cardiac indications. Furthermore, heterogeneous populations were enrolled in our study, including cardiac, respiratory, and ECPR cases as well as septic shock patients; this might obscure the effect of our ECMO team, as mortalities of septic shock and trauma patients treated with ECMO are reportedly high [2, 4, 14]. (2) Skill level of the staff: As described above, many attending physicians and medical staff members had an experience of > 20 years in performing ECMO, before the team’s establishment. This might be a reason why formal commencement of the multidisciplinary ECMO team did not dramatically change the skills of the staff members in our hospital.

All these factors could explain the unchanged survival rate in the PRE and POST groups. Even though the rate of survival has not increased, the number of ECMO cases has been continually increasing, as shown in Fig. 1; accordingly, the number of patients discharged from the hospital after ECMO treatment increased. Since number of ECMO case is increasing all over the world, according to accumulated evidences of clinical usefulness in patients with a variety of conditions [1–3], increased number of ECMO cases in this study might not be directly affected by ECMO team establishment. However, after launch of ECMO team, all staffs in our institution had become easily able to propose ECMO treatment according to the clearly described manuals. In addition, the announcement of ECMO team establishment to other departments and hospitals in the region might affect increased number of patients being introduced to our team and, thus, lead to appropriate inclusion of patients.

Moreover, the team-based approach influenced the mechanical ventilator settings during ECMO treatment. Mechanical ventilation strategy during ECMO in our institution was based on the protocol of the CESAR trial [1], which consists of a pressure-controlled ventilation mode under the peak pressure of < 25 cmH_2_O, PEEP of 10 cmH_2_O, and inspired oxygen fraction of 0.3. Comparison between PRE and POST groups revealed that PEEP at 6 h after ECMO initiation was higher, suggesting that the mechanical ventilator protocol was appropriately applied in more patients. Although these changes might have been affected by the creation of the ECMO team, the clinical outcomes such as ventilator-free days and mortality remained unaffected. These findings were consistent with those of a report by Serpa Neto et al., where none of the values for PEEP, tidal volume, plateau pressure, and respiratory rate during ECMO were associated with in-hospital mortality [15]. Significantly higher PaO2 at 6 h after ECMO initiation was observed in POST group, albeit PaO2 before ECMO was not different between two groups. PaO2 in patients with extremely low cardiac function treated with ECMO reflected amount of blood flow provided by ECMO, while PaO2 before ECMO initiation was mainly influenced by baseline condition of patients. Therefore, higher PaO2 in the POST group might suggest two possibilities: (1) more appropriate ECMO management after ECMO team formation and (2) higher severity of patients, whose cardiac function was worse, in the POST group than in the PRE group.

However, the effective recording of data during ECMO initiation was significantly improved following the establishment of the ECMO team. Additionally, an increasing number of ECMO cases combined with improving the quality of data collection can gain more experience for our ECMO team, leading to improved clinical results in the future.

The present study has several limitations. The main limitation is its retrospective nature and a relatively small sample size, which might lead to statistical error and allow unrecognized bias. In particular, retrospective design limited accurate data collection especially in PRE-ECMO team group, thus comparison of clinically important points, such as complication rate, was not possible. Furthermore, the time point of ECMO team implementation was not practical but formal on paper, since before the launch of the team many of the team’s physicians and staff members played similar roles in the management of ECMO, training of staff members, and in collection of data. Finally, the inclusion and exclusion criteria in this study resulted in the enrollment of mixed type of patients, leading to conflicting results. Specifically, this cohort mainly consisted of ECPR cases, those might hardly benefit from team approach. Further studies are warranted to reveal whether multidisciplinary ECMO team approach improves clinical outcomes.

## Conclusion

Following the establishment of an ECMO team, the survival rate of patients treated with ECMO, ECMO duration, and length of hospital stay were not improved. However, after the group was formally constituted, the number of cases treated with ECMO increased and the clinical data collection rate was improved. More effective ECMO treatment may be achieved by additional investigation of the relevant clinical factors, guided by an improved quality of clinical data collection. Therefore, further study is warranted.

## Abbreviations

ABG: Arterial blood gas
ACT: Activated clotting time
ARDS: Acute respiratory distress syndrome
CPR: Cardiopulmonary resuscitation
ECMO: Extracorporeal membrane oxygenation
ECPR: Extracorporeal cardiopulmonary resuscitation
IQR: Interquartile ranges
MAP: Mean arterial blood pressure
V-A: Veno-arterial
V-V: Veno-venous
